# Corrigendum: A Glycosylphosphatidylinositol-Anchored α-Amylase Encoded by *amyD* Contributes to a Decrease in the Molecular Mass of Cell Wall α-1,3-Glucan in *Aspergillus nidulans*

**DOI:** 10.3389/ffunb.2022.872790

**Published:** 2022-03-07

**Authors:** Ken Miyazawa, Takaaki Yamashita, Ayumu Takeuchi, Yuka Kamachi, Akira Yoshimi, Yuto Tashiro, Ami Koizumi, Makoto Ogata, Shigekazu Yano, Shin Kasahara, Motoaki Sano, Youhei Yamagata, Tasuku Nakajima, Keietsu Abe

**Affiliations:** ^1^Laboratory of Applied Microbiology, Department of Microbial Biotechnology, Graduate School of Agricultural Science, Tohoku University, Sendai, Japan; ^2^Laboratory of Filamentous Mycoses, Department of Fungal Infection, National Institute of Infectious Diseases, Tokyo, Japan; ^3^Laboratory of Environmental Interface Technology of Filamentous Fungi, Graduate School of Agriculture, Kyoto University, Kyoto, Japan; ^4^ABE-Project, New Industry Creation Hatchery Center, Tohoku University, Sendai, Japan; ^5^Faculty of Food and Agricultural Sciences, Fukushima University, Fukushima, Japan; ^6^Department of Biochemical Engineering, Graduate School of Engineering, Yamagata University, Yonezawa, Japan; ^7^Food Microbiology Unit, School of Food and Agricultural Sciences, Miyagi University, Sendai, Japan; ^8^Genome Biotechnology Laboratory, Kanazawa Institute of Technology, Hakusan, Japan; ^9^Department of Applied Life Science, The United Graduate School of Agricultural Science, Tokyo University of Agriculture and Technology, Fuchu, Japan; ^10^Department of Microbial Resources, Graduate School of Agricultural Science, Tohoku University, Sendai, Japan

**Keywords:** cell wall, filamentous fungi, *Aspergillus nidulans*, glycosylphosphatidylinositol-anchored protein, α-amylase, α-1, 3-glucan

In the original article, “the funder the Japan Society for Promotion of Science (JSPS) KAKENHI, 20H02895” to Keietsu Abe was not included. The corrected **Funding** statement appears below.

**Funding**

This work was supported by the Japan Society for Promotion of Science (JSPS) KAKENHI Grant Nos. 26292037 (KA), 18K05384 (KA), 20H02895 (KA) and 20K22773 (KM), and a Grant-in-Aid for JSPS Fellows Grant No. 18J11870 (KM). This work was also supported by the Institute for Fermentation, Osaka (Grant No. L-2018–2–014) (KA) and by the project JPNP20011 (KA), which is commissioned by the New Energy and Industrial Technology Development Organization (NEDO).

The authors apologize for this error and state that this does not change the scientific conclusions of the article in any way. The original article has been updated.

## Publisher's Note

All claims expressed in this article are solely those of the authors and do not necessarily represent those of their affiliated organizations, or those of the publisher, the editors and the reviewers. Any product that may be evaluated in this article, or claim that may be made by its manufacturer, is not guaranteed or endorsed by the publisher.

